# Platypnea–orthodeoxia syndrome associated with COVID-19 pneumonia: a case report

**DOI:** 10.1186/s40981-021-00471-7

**Published:** 2021-08-31

**Authors:** Takuo Hoshi, Yu Tadokoro, Masaru Nemoto, Junya Honda, Shihori Matsukura

**Affiliations:** 1 Department of Anesthesiology and Critical Care Medicine, Ibaraki Clinical Education and Training Center, University of Tsukuba, Koibuchi 6528, Kasama, Ibaraki, 309-1793 Japan; 2 Department of Vascular and Endovascular Surgery, Ibaraki Prefectural Central Hospital, Kasama, Japan; 3 Department of Cardiovascular Medicine, Ibaraki Prefectural Central Hospital, Kasama, Japan; 4 Department of Respiratory Medicine, Ibaraki Prefectural Central Hospital, Kasama, Japan

**Keywords:** Platypnea–orthodeoxia syndrome, COVID-19 pneumonia, Positional change

## Abstract

**Background:**

Platypnea–orthodeoxia syndrome (POS) is a rare clinical condition characterized by respiratory distress and/or hypoxia developing in the sitting/upright position, which is relieved in the recumbent position. This syndrome is known to have an intracardiac shunt as its primary etiology. Here, we report the case of a patient who was found to have POS without an intracardiac shunt while recovering from coronavirus disease (COVID-19) pneumonia.

**Case presentation:**

A 73-year-old woman was diagnosed with severe COVID-19 pneumonia and was managed according to our institutional protocol. Although her oxygenation improved at rest, oxygen saturation dropped to lower than 80% when she was in the sitting position. She had no patent foramen ovale or other intracardiac shunts. She showed gradual improvement and was discharged under home oxygen therapy 28 days after admission.

**Conclusions:**

This report highlights the importance of continuous bedside monitoring of pulse oximetry during positional changes, even if it is stable at rest, in patients with moderate to severe COVID-19.

## Background

Platypnea–orthodeoxia syndrome (POS) is a rare condition first described by Burchell et al. [1] in 1949, which causes hypoxemia when sitting or standing and improves in the supine position. The common etiology of this syndrome is the presence of a right–left shunt, such as a patent foramen ovale (PFO) or an atrial septal defect (ASD) [2]. Here, we report the case of a patient who was found to have POS without an intracardiac shunt during recovery from coronavirus disease (COVID-19) pneumonia. Written patient consent was obtained for the publication of the report, which was prepared according to the CARE guidelines.

## Case presentation

A 73-year-old woman (height, 157 cm; weight, 43 kg) visited a physician 7 days before admission to our hospital for dizziness, loss of appetite, and fatigue, but no specific issues could be determined, and the patient returned home without receiving treatment. On the day of admission, she revisited the physician because she had a fever of 38°C and dyspnea. Her oxygen saturation was 86% in ambient room air, and nasopharyngeal swab polymerase chain reaction for SARS-Cov-2 revealed that she had COVID-19. The patient was transferred to our hospital under oxygen administration for respiratory management.

When the patient arrived at our hospital, her oxygen saturation was 87% (blood pressure, 152/68 mmHg; heart rate, 73 bpm) with oxygen administered at 2 L/min via a nasal cannula. We changed the oxygen administration method to OxyMask^TM^ (Southmedic Inc., Barrie, ON, Canada) at 7 L/min, and her oxygen saturation increased to 92%. Subsequently, she was admitted to the intensive care unit. Although she had no underlying conditions that would increase her risk for severe COVID-19, shortly after admission, her oxygen saturation dropped to approximately 90% and her respiratory rate increased to 40/min. We decided to start high-flow nasal cannula therapy at 50 L/min, FIO2 50%.

**Fig. 1:**
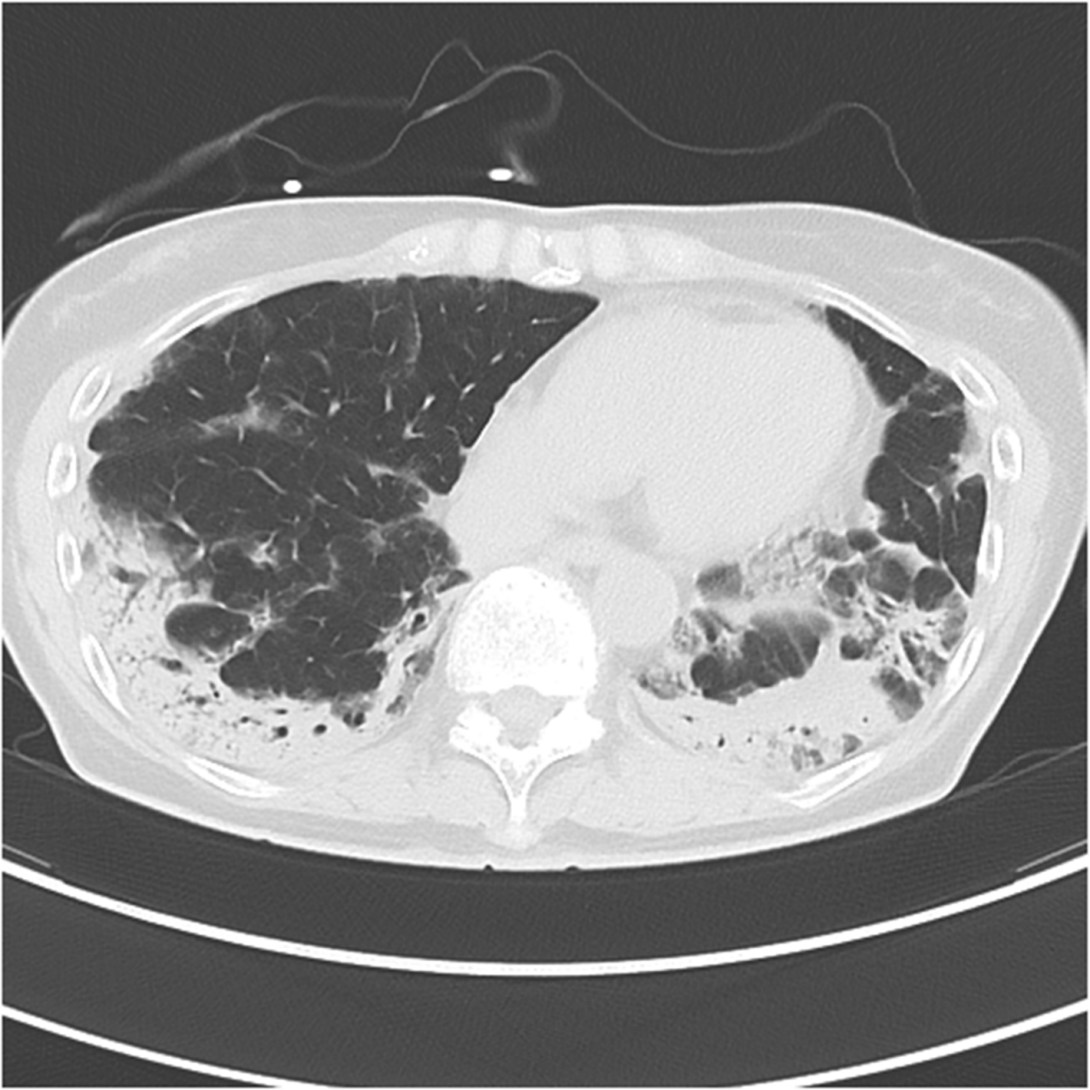
Initial chest computed tomography (CT) showed bilateral and peripheral predominant consolidation and air bronchogram

Her hemogram showed a white blood cell count of 7400/μl and hemoglobin 12.4 g/dl. Her blood biochemistry tests only showed mild elevation in transaminases (glutamic oxaloacetic transaminase, 46 U/l; glutamic pyruvic transaminase, 36 U/l), and her renal function tests and electrolytes were within the normal limits. Arterial blood gas analysis performed before high-flow oxygen therapy was suggestive of type 1 respiratory failure (pH, 7.496; PO_2_, 52.2 mmHg; PCO_2_, 28.5 mmHg). Initial chest computed tomography (CT) (Fig. 1) showed bilateral and peripheral predominant consolidation and an air bronchogram.

She was managed according to our institutional protocol (inhalational oxygen concentration, steroids, anticoagulation, tocilizumab, rehabilitation) and maintaining oxygen saturation 92%. Although she temporarily needed an FIO_2_ of 0.8 to maintain her peripheral oxygen saturation, we did not intubate her because she did not exert effort during ventilation, she strongly preferred not to be intubated, and her only symptom was lightheadedness when standing. On the 11th day of hospitalization, although her oxygen saturation decreased with light exertion but stabilized at rest in the supine position, we changed the oxygen administration method to OxyMask^TM^ 8 L/min. Although oxygen saturation was maintained when the patient was at rest and in the supine position, it dropped to lower than 80% when the patient was in the sitting position after, for instance, moving to a portable toilet. Moreover, more than 30 min was needed for oxygen saturation to increase even after starting high-flow oxygen therapy with fraction of inspiratory oxygen 0.5 or higher. However, SpO2 rapidly recovered when the patient was repositioned to the supine position. We suspected the presence of a right–left shunt, which increased with change in position, and asked the cardiologist to search for a PFO or ASD. We performed contrast-enhanced CT and echocardiography but could not find an intracardiac shunt. Afterward, although her oxygen saturation slightly decreased when she sat or stood, her oxygenation slowly improved, and she was discharged under home oxygen therapy with 0.5 L/min via a nasal cannula 28 days after admission.

## Discussion

We reported the case of a patient with POS secondary to COVID-19 pneumonia. POS is a rare syndrome characterized by dyspnea and hypoxia in the sitting or upright position from a recumbent position and fall in oxygen saturation by more than 5% [3]. The mechanism of POS can be classified based on intracardiac abnormalities, extracardiac abnormalities, and miscellaneous etiologies [2, 3], and a PFO is the most commonly reported etiology of this syndrome, and few cases related to lung parenchymal disease have been reported [3].

Reported causes of POS include parenchymal lung disease, interstitial lung disease, and adult respiratory distress syndrome [3, 4], and POS caused by COVID-19 was first reported in 2020 [5]. In patients with COVID-19, posterior and lower zone parenchymal involvement and gravitational shunting of blood to the lower zones to wasting of ventilation are common [6]. Wasting of ventilation is also exaggerated in the presence of microthrombi and microangiopathy observed in severe COVID-19 [7]. In the present case, contrast-enhanced CT showed no obvious pulmonary emboli, but the D-dimer was slightly high at 1.4 μg/ml at admission, indicating the possible presence of a microthrombus. Patients with COVID-19 reportedly have varying degrees of pulmonary fibrosis and lung dysfunction, necessitating careful monitoring [8]. Lung lesions caused by COVID-19 often occur in the subpleural area of the lower lobes of both lungs [9], and in our patient, the basal part of both lungs was predominantly affected by the interstitial pneumonia’s fibrotic evolution. As gravity promotes blood flow more to the basilar areas of the lung, the apical lung zones show increased dead space ventilation. Lower lung-predominant parenchymal disease may worsen this physiological ventilation–perfusion mismatch, resulting in POS.

Furthermore, in our patient, the physiological shunt as well as the wasted ventilation was amplified by the disproportionate reduction of ventilation with respect to the reduced perfusion when shifting from recumbent to the sitting or standing position. Her physiological shunt and waste ventilation were improved by rehabilitation and inflammation improvement, and the patient could be discharged under home oxygenation therapy. Although COVID-19 pneumonia predominantly affects the basal segments of both lungs, it remains unclear why POS is not observed in all COVID-19 pneumonia cases. In our case, D-dimer was only measured at the time of admission, but we speculate that the physiology of the vascular bed may have significantly changed because of the progression of the pulmonary microembolism and subsequent vascular changes; reviewing the blood test schedule may help in early detection.

## Conclusions

POS is a rare syndrome and should be considered in the differential diagnosis of dyspnea and refractory hypoxemia during rehabilitation of moderate to severe COVID-19 pneumonia. It is crucial to diagnose and determine its etiology to provide appropriate management.

## Data Availability

Not applicable.

## Abbreviations

ASD: Atrial septal defect
CT: Computed tomography
FIO2: Inhalational oxygen fraction
PCO2: Partial pressure of arterial carbon dioxide
PFO: Patent foramen ovale
pH: Potential of hydrogen
PO2: Partial pressure of arterial oxygen
POS: Platypnea-orthodeoxia syndrome
